# NLRP3 ablation enhances tolerance in heat stroke pathology by inhibiting IL-1β-mediated neuroinflammation

**DOI:** 10.1186/s12974-021-02179-y

**Published:** 2021-06-06

**Authors:** Zi-Teng Zhang, Xiao-Lei Gu, Xin Zhao, Xian He, Hao-Wei Shi, Kun Zhang, Yi-Ming Zhang, Yi-Nan Su, Jiang-Bo Zhu, Zhi-Wei Li, Guo-Bao Li

**Affiliations:** 1 Department of Hepato-Biliary Surgery, Shenzhen Third People’s Hospital, The Second Affiliated Hospital, Southern University of Science and Technology, No.29 Bulan Road, Longgang District, Shenzhen, 518055 China; 2 Department of Pharmacy, The Affiliated Tumor Hospital of Zhengzhou University, Zhengzhou, 450008 China; 3 School of Pharmacy, Dali University, Dali, 671000 China; 4 Fifth Medical Center of PLA General Hospital, Beijing, 100000 China; 5 Department of Neurosurgery, Hebei Provincial People’s Hospital, Shijiazhuang, 050051 China; 6 Department of Health Toxicology, Faculty of Navy Medicine, Navy Medical University, Shanghai, 200433 China; 7 Department of Lung Disease, Shenzhen Third People’s Hospital, The Second Affiliated Hospital, Southern University of Science and Technology, No.29 Bulan Road, Longgang District, Shenzhen, 518055 China

**Keywords:** NLRP3 inflammasome, Heatstroke, Neuroinflammation, IL-1β

## Abstract

**Background:**

Patients with prior illness are more vulnerable to heat stroke-induced injury, but the underlying mechanism is unknown. Recent studies suggested that NLRP3 inflammasome played an important role in the pathophysiology of heat stroke.

**Methods:**

In this study, we used a classic animal heat stroke model. Prior infection was mimicked by using lipopolysaccharide (LPS) or lipoteichoic acid (LTA) injection before heat stroke (LPS/LTA 1 mg/kg). Mice survival analysis curve and core temperature (T_C_) elevation curve were produced. NLRP3 inflammasome activation was measured by using real-time PCR and Western blot. Mice hypothalamus was dissected and neuroinflammation level was measured. To further demonstrate the role of NLRP3 inflammasome, *Nlrp3* knockout mice were used. In addition, IL-1β neutralizing antibody was injected to test potential therapeutic effect on heat stroke.

**Results:**

Prior infection simulated by LPS/LTA injection resulted in latent inflammation status presented by high levels of cytokines in peripheral serum. However, LPS/LTA failed to cause any change in animal survival rate or body temperature. In the absence of LPS/LTA, heat treatment induced heat stroke and animal death without significant systemic or neuroinflammation. Despite a decreased level of IL-1β in hypothalamus, *Nlrp3* knockout mice demonstrated no survival advantage under mere heat exposure. In animals with prior infection, their heat tolerance was severely impaired and NLRP3 inflammasome induced neuroinflammation was detected. The use of *Nlrp3* knockout mice enhanced heat tolerance and alleviated heat stroke-induced death by reducing mice hypothalamus IL-1β production with prior infection condition. Furthermore, IL-1β neutralizing antibody injection significantly extended endotoxemic mice survival under heat stroke.

**Conclusions:**

Based on the above results, NLRP3/IL-1β induced neuroinflammation might be an important mechanistic factor in heat stroke pathology, especially with prior infection. IL-1β may serve as a biomarker for heat stroke severity and potential therapeutic method.

## Introduction

Heat stroke is characterized clinically by severe hyperthermia and defined as a core temperature of > 40.5 °C. The manifestations include headache, sweating, tachycardia, and lightheadedness, and it might soon progress to muscle cramps, oliguria, hypotension, syncope, confusion, and coma if the core temperature does not reduce immediately [1]. Many studies implicated systemic inflammatory response syndromes (SIRS) and central nervous system (CNS) collapse in this disorder [2, 3].

Body’s tolerance to heat varies widely among individuals. A mild illness develops in response to heat in some people, whereas in others, the condition progresses to heat stroke. Widely accepted predisposing factors consist of prolonged or intense exercise, lack of heat acclimatization, sleep deprivation, dehydration, alcohol abuse, drug abuse, chronic inflammation, febrile illness, and older age [4]. Among all the factors, pre-existing illness caused by Gram-negative (G-) bacteria (LPS) or Gram-positive (G+) bacteria (LTA) was believed to play a crucial role in heat tolerance impairment and increase susceptibility to heat stroke [5]. The symptoms in heat stroke and infection are similar, such as hyperthermia, dehydration, neurological abnormalities, and inflammation. Prior infection is known to impair heat tolerance, potentially by pre-inducing a febrile and immune compromised state [6]. Pro-inflammatory cytokines including IL-1β, IL-6, and TNF-α are elevated in both infection and heat stroke, suggesting infection might prime a latent immune response that is synergistically driven by heat challenge and promoted severe pathology [7]. During the 1995 heat wave in Chicago, a high percentage (57%) of classic heatstroke patients had evidence of infection on admission and the mortality rate was as high as 21% among these patients during acute hospitalization [8]. Multiple ideas have been put forth as to how pre-existing endotoxemia compromises heat tolerance and the role of thermal regulation, and deregulation is indeed increasingly appreciated. However, the underlying pathophysiological processes are still controversial.

CNS maintains body temperature during environmental temperature challenges and alters body temperature during the inflammatory response and behavioral states in response to declining energy homeostasis [9, 10]. Thermoregulatory circuitry in the hypothalamus exerts heat defense mechanisms by serious effectors to increase heat loss, such as cardiac output increase, vasodilation, peripheral blood flow increase, and sweating [11]. Therefore, CNS dysfunction, in particular hypothalamus thermoregulatory network dysfunction, was identified as an initial and driving factor during heat stroke [12].

In the past decades, a variety of studies have been indicated that the pathophysiologic responses to heatstroke are associated with the systemic inflammatory response syndrome, which might implicate gut-derived endotoxin [13, 14]. Inflammatory cytokines such as interleukin-1 beta (IL-1β), interleukin-6 (IL-6), and tumor necrosis factor-α (TNF-α) seem to be of great significance in the pathogenesis of heatstroke. Elevated concentrations of these cytokines have been found in experimental animals and patients with heat stroke [15–17]. NACHT, LRR, and PYD domain-containing protein 3 (NALP3) is a cytosolic pattern recognition receptor and could form a caspase-1 activating complex (NLRP3 inflammasome) with the adaptor ASC protein PYCARD, which then regulates the cleavage and maturation of IL-1β and IL-18 [18].

However, to our knowledge, the role of NLRP3 inflammasome in the CNS during heatstroke has also not been fully investigated before. This study aimed to investigate the effect of CNS NLRP3 inflammasome in heat stroke in endotoxemic mice. It was hypothesized that pre-existing LPS compromised heat tolerance through the activation of NLRP3 inflammasome, and *Nlrp3* knockout or against IL-1β therapy during heat exposure could increase heat tolerance.

## Methods

### Reagents

Lipopolysaccharide (LPS) from Escherichia coli O111:B4 (catalog: L2630-100MG) was purchased from Sigma-Aldrich (St. Louis, MO, USA); lipoteichoic acid (LTA) was from *Staphylococcus aureus* (L2515, Sigma-Aldrich, St Louis, MO, USA); anti-mouse NLRP3 antibody (AG-20B-0014) was purchased from AdipoGen Corp. (San Diego, CA); anti-mouse caspase-1 p10 antibody (sc-514) and anti-mouse β-actin antibody (sc-47778) were purchased from Santa Cruz Biotechnology, Inc. (CA. USA); anti-mouse IL-1β antibody was bought from Cell Signaling Technology (Beverly, MA). The reagents listed above were prepared and used according to the manufacturer’s instructions.

### Animals

Wild type (WT) C57BL/6 male mice, 5–7 weeks age, were purchased from Super-B&K Laboratory Animal Corp. Ltd, Shanghai, China. C57BL/6 *Nlrp3* knockout mice were obtained from the Model Animal Research Centre of Nanjing University (AAALAC accredited) as previously reported [19]. The animals were housed in Specific Pathogen Free (SPF) animal facility for at least 1 week under a 12-h light/dark cycles (8:00–20:00 light and 20:00–8:00 dark). The mice were acclimatized to room temperature at 22 ± 2 °C under relative humidity of 50 ± 5%, with free access to water and standard laboratory chow ad libitum.

### LPS/LTA injection and heat treatment conditions

In vivo, the methods to induce heat stroke in mice were carried out as described previously with a small modification because of different animal strain [2]. The mice were moved to the Climatic & Environmental Simulation Center in the Navy Medical University where whole-body heating (WBH) maintains a condition of 41.2 °C, and relative humidity 50 ± 5% was used in an environment-controlled cabin. The pre-existing LPS/LTA groups were intraperitoneally injected with 1 mg/kg 4 h before heat stroke experiment. At the same time, normal saline involved in the control. The experiment started at 09:30 AM; core temperature (T_C_) was monitored in every 15 min using a digital thermometer (ALC-ET06, Shanghai Alcott Biotech Co., China) which was inserted 2 cm into the rectum. The mice underwent the same experimental procedure as the heat stroke mice with a cabin temperature of 22 ± 2 °C. The control group with relative humidity of 50 ± 5% was used as no heat ones in the experiment. Mice with the IL-1β neutralizing antibody (Cat: AF-401-NA, R&D Systems, Minneapolis, MN) were injected intravenously 30 min before heat stroke in two dosages which were 0.04 μg/g and 0.2 μg/g body weight. All animal experiments used in this study were approved by the Institutional Animal Care and Use Committee of Navy Medical University (No. 20200075, Shanghai, China), and all procedures were performed in compliance with the Guide line for Care and Use of Laboratory Animals published by the National Institutes of Health, USA.

### Assessment of the heat tolerance

The assessment of the heat tolerance consisted of two parts. First, the moment at which core temperature reached 42 °C was set as the time point for the onset of heat stroke [20]. The time duration for T_C_ to increase from resting to 42 °C was collected. Second, mice survival time under above heat treatment was collected and Kaplan-Meier curve was drawn. The following four groups were created in vivo studies: control/no heat, control/heat, LPS/no heat, and LPS/heat groups (control/no heat, control/heat, LTA/no heat, and LTA/heat groups).

### Brain and serum sample collection

The mice were anesthetized by intraperitoneal injection of sodium pentobarbital (50 mg/kg body weight) at heat stroke time point. The mice were then sacrificed and whole brain was rapidly extracted and placed on icebox. The diencephalon region (mainly the hypothalamus) was quickly dissected and then frozen in liquid nitrogen for further assessment. Blood samples were harvested from abdominal aorta, and the serum was separated by centrifugation at 3000 rpm for 30 min at 4 °C (Eppendorf 5801R centrifuge, Germany), and then stored at − 80 °C for biochemical and enzyme-linked immunosorbent assay (ELISA) analysis.

### Biochemical and cytokines measurement

The levels of LDH were determined by HITACHI 7080 automated analyzer (Japan). Commercial ELISA kits were used for the measurement of tumor necrosis factor alpha (TNF-α), interleukin-6 (IL-6), and IL-1β (R&D system) using a SpectraMax M2e Microplate Spectrophotometer (Molecular Devices, CA, USA) according to the manufacturer’s instruction.

### Real-time PCR analysis of NLRP3 and pro-IL-1β mRNA

Total RNA was isolated by using Trizol reagent (Life Technologies, USA). Reverse transcription was performed with 1μg total RNA using Transcriptor First Strand cDNA Synthesis Kit (Roche Ltd, Swiss). Total of 2 μl first-strand cDNA solution was used for real time RT-PCR in combination with Fast Start Universal Probe Master (ROX) in a final volume of 20 μl. All experiments were run in triplicate and underwent 40 amplification cycles by using Applied Biosystems 7500 system (Life Technologies Corporation., USA). The primers used for RT-PCR were listed in Table 1. The threshold cycle (CT) of target product was normalized to internal standard GADPH and calculated by using the comparative cycle threshold (^ΔΔ^Ct) method.

**Table 1:** Primer used for real-time PCR in this study

Genes	Forward primer(5^,^→3^,^)	Reverse primer(5^,^→3^,^)
NLRP3	ACCAGCCAGAGTGGAATGAC	ATGGAGATGCGGGAGAGATA
Pro-IL-1β	CTCACAAGCAGAGCACAAGC	TCCAGCCCATACTTTAGGAAGA
GADPH	GTGTTTCCTCGTCCCGTAGA	AATCTCCACTTTGCCACTGC

### Western Blot analysis

For Western Blot analysis, mice diencephalon tissue was homogenized and the protein concentrations were measured as we described previously [15]. Cleared homogenates were separated by 10% SDS-PAGE, transferred onto PVDF membranes, and then blocked for 2 h at room temperature with 5% non-fat dried milk and incubated with anti-NLRP3 Ab, anti-caspase-1 Ab, and anti-IL-1β Ab and then exposed with an Amersham Imager 600. The band intensity values of the target proteins were normalized to that of β-actin.

### Immunohistochemistry analysis of IL-1β

Formalin-buffered diencephalon tissue samples were embedded in paraffin wax and subsequently cut into 2-μm slices, using a rotary microtome. The sections were subjected to an antigen retrieval procedure and then rinsed in distilled water, washed in 0.1 M PB for 10 min, and incubated in a blocking buffer (0.5% goat serum S-1000, Vector Labs, in 0.1 M PB) at room temperature for 1 h. They were then incubated overnight at 4 °C in a humidified chamber with primary anti-IL-1β ab with blocking buffer. The rest procedures were conducted according to manufacturer’s protocols (Vectastain ABC kit elite DK-6100 standard, Vector Labs). Finally, sections were washed, dehydrated, and mounted with non-aqueous mounting medium (Permount, Fisher) to perform cell counts. As a negative control, sections were incubated without primary antibodies. The sections were examined using an Olympus AX70 microscope. For the intensity, the quantitation and score were conducted as previously reported [21].

### Statistical analysis

The results are measured and expressed as the mean ± sem. One-way ANOVA was used to detected statistical significance among group means, and Dunnett’s test analysis was used to compare specific two groups when ANOVA showed significant difference. The comparisons made between two groups were evaluated using Student’s independent t test. In survival curve, the comparison was conducted by using log-rank test method. All statistical analyses were performed using SPSS 21 software. *P* < 0.05 was considered to be statistically significant.

## Results

### Effects of LPS/LTA and heat treatment on heat tolerance in mice

The tolerance to heat in mice was assessed in two parts as described. In order to comprehensively illustrate the effect of LPS and heat treatment, animals were divided into four groups: control/no heat, control/heat, LPS/no heat, and LPS/heat group in vivo.

**Fig. 1:**
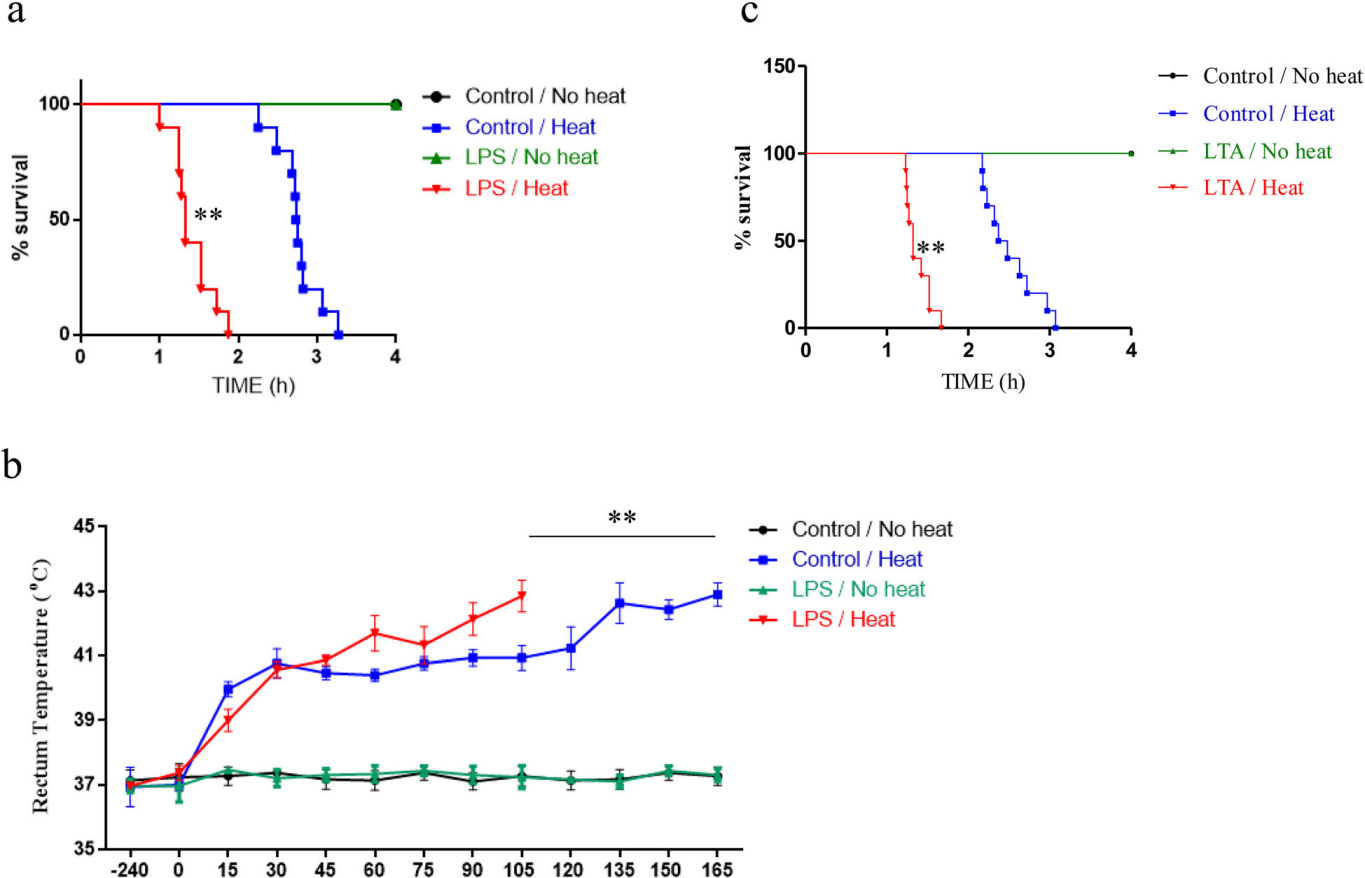
Prior LPS compromised survival time and heat tolerance under heat exposure. The experiment was designed into four groups: control/no heat, control/heat, LPS/no heat, and LPS/heat groups. LPS treatment condition was 1 mg/kg and whole-body heating (WBH) temperature was 41.2 °C. **a** Survival curve was monitored in comparison between control/heat and LPS/heat groups. The statistical analysis was conducted by Log-rank test (*n* = 20), **p < 0.01, *p < 0.05. **b** Core temperature (T_C_) profiles of mice were monitored in every 15 min to compare among control/heat and LPS/heat groups. **c** Prior LTA compromised survival time under heat exposure. The animals were designed into four groups: control/no heat, control/heat, LTA/no heat, and LTA/heat groups. LTA treatment condition was 1 mg/kg and whole-body heating (WBH) temperature was 41.2 °C. Survival curve was monitored in comparison between control/heat and LTA/heat groups. The average time taken by core temperature to reach 42 °C was calculated as the mean ± SEM. (*n* = 5 to 10). **p < 0.01, *p < 0.05

The survival study result was drawn into K-M curve in Fig. 1a. All of the mice in control/no heat and LPS/no heat group that were not exposed to heat survived. The duration of survival was the shortest in the LPS/heat mice (84.24 ± 4.84 min), followed by the control/heat mice (165.42 ± 5.32 min) and the log-rank test found significant difference between these two groups (*p* < 0.01, Fig. 1a).

The mean time duration for T_C_ to increase from resting to 42 °C were measured in every 15 min in four groups. The T_C_ of control/no heat and LPS/no heat mice that accommodated in normal temperature remained stable in the experiment procedure. To reach 42 °C, it took 80 ± 4.3 min to reach 42 °C in LPS/heat mice, 120 ± 5.3 min in control/heat mice and the time durations were significantly different (*p* < 0.01, Fig. 1b).

In the survival test using LTA, similar to the scenario of LPS, low-dose administration (1 mg/Kg) of LTA alone was not sufficient to induce animal death; nevertheless, prior LTA significantly enhanced the fatal effect of heat stress. The survival time difference between LTA/heat mice (82.56 ± 9.01 min) and control/heat mice (150.84 ± 19.42 min) was significant (*p* < 0.01, Fig. 1c).

### Effects of LPS/LTA and heat treatment on inflammatory cytokine levels in mice

**Fig. 2:**
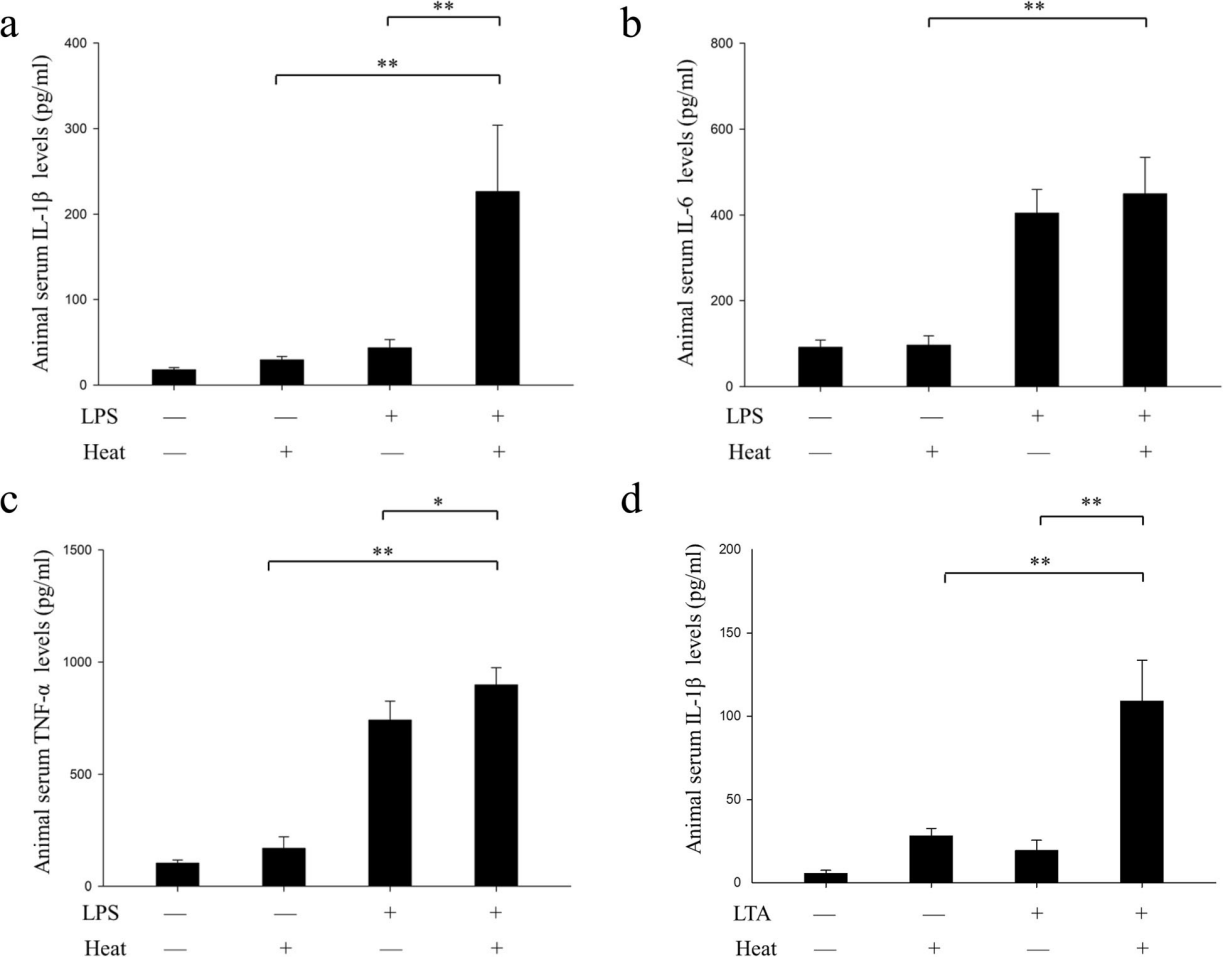
Heat treatment with prior LPS treatment increased systemic inflammatory cytokine levels in mice. Serum inflammatory cytokines were measured in four animal groups by using ELISA. **a** IL-1β was measured in four groups. **b** IL-6 was measured in four groups. **c** TNF-α was measured in four groups. **d** Heat treatment with prior LTA treatment increased systemic IL-1β cytokine levels in mice. Serum IL-1β cytokines were measured in four animal groups by using ELISA. The average number was calculated as the mean ± SEM. (*n* = 5 to 10). The average number was calculated as the mean ± SEM. (*n* = 5 to 10). **p < 0.01, *p < 0.05

Regarding animal serum inflammatory cytokine, we compared IL-1β, IL-6 and TNF-α among the four groups (Fig. 2). Assessment of IL-1β levels showed that LPS challenge combined with heat significantly increased the production of serum IL-1β when compared to either control/heat group (226.16 ± 78.08 pg/ml vs 29.50 ± 3.96 pg/ml, *p* < 0.01, Fig. 2a) or LPS/no heat group (226.16 ± 78.08 pg/ml vs 43.49 ± 9.53 pg/ml, *p* < 0.01, Fig. 2a). LPS treatment alone did increase serum IL-1β level compared with control/no heat group, but the difference failed to alter mice survival time (Fig. 1). Heat treatment alone also increased serum IL-1β level compared with control/no heat group from 17.87 ± 2.51 pg/ml to 29.50 ± 3.96 pg/ml.

As for the result of IL-6, LPS/heat treatment significantly increased serum IL-6 levels when compared to control/heat group (*p* < 0.01, Fig. 2b), but we detected no significant difference in comparison with LPS/no heat group (448.89 ± 85.36 pg/ml vs 404.66 ± 54.78 pg/ml, *p* = 0.31, Fig. 2b).

In the measurement of TNF-α, the result indicated that LPS/heat treatment significantly increased serum TNF-α production when compared with control/heat group (898.60 ± 76.72 pg/ml vs 740.92 ± 8531 pg/ml, *p* < 0.05, Fig. 2c). In comparison with LPS/no heat group, similar difference was found (898.60 ± 76.72 pg/ml vs 169.17 ± 51.63 pg/ml, *p* < 0.01, Fig. 2c).

In the study of LTA, neither LTA nor heat alone could induce IL-1β secretion, but their combination could release large amount of IL-1β. There was a significant difference between control/heat group and LTA/heat group (28.07 ± 4.51 pg/ml vs 109.01 ± 24.63 pg/ml, *p* < 0.01, Fig. 2d). The difference between LTA/no heat group and LTA/heat group was also significant (19.20 ± 6.21 pg/ml vs 109.01 ± 24.63 pg/ml, *p* < 0.01, Fig. 2d).

### Heat stress promoted NLRP3 inflammasome activation in murine hypothalamus with LPS pretreatment

We further test whether NLRP3 inflammasome was activated in vivo under heat. It has been suggested that hypothalamus was the central nerve region in temperature regulation [22]. Its damage plays a crucial role in temperature disturbances, such as fever or hypothermia [23]. So, mice hypothalamus was dissected and prepared for RT-PCR and Western Blot analysis.

**Fig. 3:**
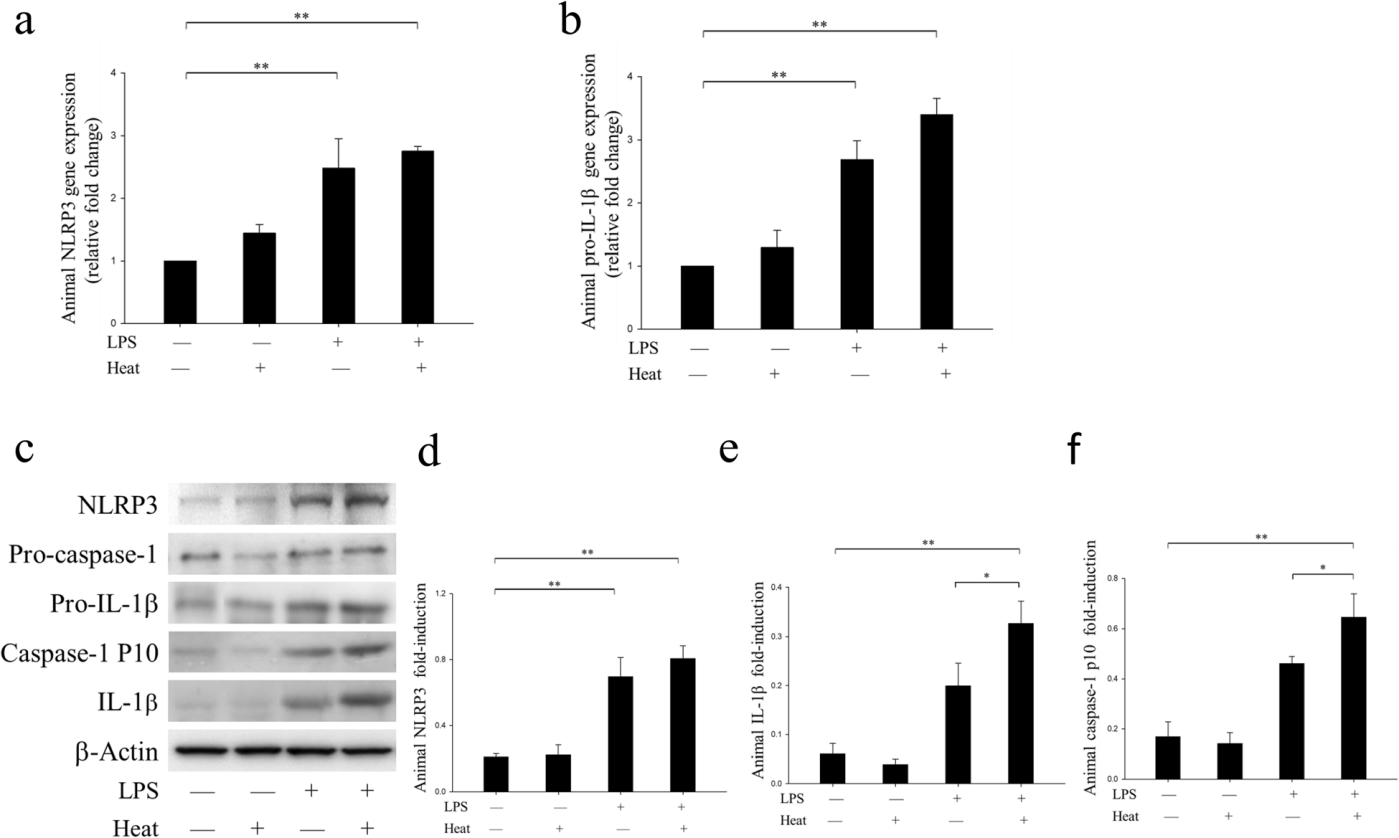
LPS plus heat exposure promoted NLRP3 inflammasome activation in murine hypothalamus by using Real time-PCR, Western Blot, and immunofluorescence staining assay. Hypothalamus was chosen to be the target tissue and four groups were analyzed accordingly. **a** Real time-PCR analysis of NLRP3 mRNA. **b** Real time-PCR analysis of pro-IL-1β mRNA. **c** Western Blot result of NLRP3-related proteins. **d** Band intensity analysis of NLRP3protein. **e** Band intensity analysis of IL-1βprotein. **f** Band intensity analysis of caspase-p10 protein. Band intensities were quantified by ImageJ software and the values of the target proteins were normalized to that of β-actin. The threshold cycle (CT) of target product was normalized to internal standard GADPH and calculated by using the comparative cycle threshold (^ΔΔ^Ct) method. Band intensities were quantified by Image J software and the values of the target proteins were normalized to that of β-actin. The results were expressed as the mean ± SEM. (*n* = 5 to 10). **p < 0.01, *p < 0.05

NLRP3 protein transcription and translation are crucial for the formation and activation of NLRP3 inflammasome [24]. Therefore, we measured the effect of LPS and heat on NLRP3 expression on both mRNA and protein levels. NLRP3 and pro-IL-1β mRNA levels were measured by using RT-PCR. As shown in Fig. 3a and b, LPS/no heat mice showed significant increased level of both NLRP3 (*p* < 0.01, Fig. 3a) and pro-IL-1β (*p* < 0.01, Fig. 3b) in comparison with control/no heat mice. Nevertheless, control/heat mice failed to demonstrate significant increase compared with control/no heat mice. This result suggested that heat alone wasn’t enough to increase NLRP3 and pro-IL-1β mRNA transcription. As expected, LPS/heat mice exhibited significant increase in NLRP3 and pro-IL-1β mRNA levels in the hypothalamus when compared with that of the control/no heat mice (*p* < 0.01).

We then performed Western Blot to detect the target protein: NLRP3, caspase-1 p10 and IL-1β. Consistent with the result of NLRP3 mRNA data, LPS/no heat mice showed remarkable increased NLRP3 protein level when compared with control/no heat mice, while heat alone had no such effect (*p* < 0.01, Fig. 3c, d). By contrast, heat treatment alone was unable to cause such an effect. Caspase-1 p10 and IL-1β were the main products of NLRP3 inflammasome, once NLRP3 inflammasome was assembled, pro-IL-1β was cleaved into active IL-1β by caspase-1 p10, and so the protein levels of caspase-1 p10 and IL-1β were sensitive markers for NLRP3 inflammasome activation. As shown in Fig. 3e, though LPS administration alone could be some extent to induce L-1β production, LPS/heat mice illustrated significant IL-1β to increase in comparison with LPS/no heat mice (*p* = 0.03, Fig. 3e). The test of caspase-1 p10 showed similar result (*p* = 0.02, Fig. 3f). These results together provided a strong evidence of NLRP3 inflammasome assembly in LPS/heat mice.

### *Nlrp3* knockout failed to reduce mice death caused by heat treatment alone

**Fig. 4:**
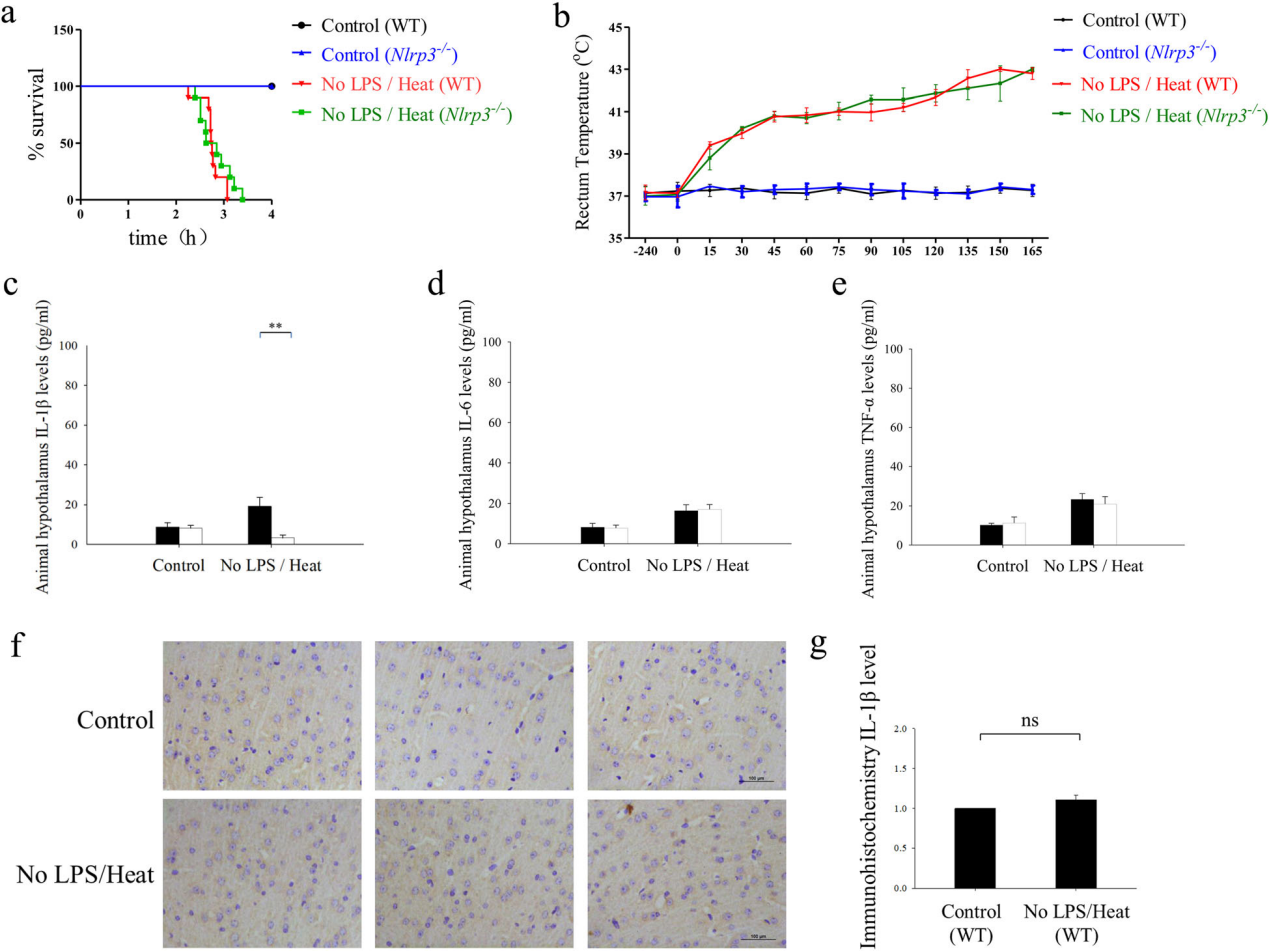
*Nlrp3* knockout failed to reduce mice death which was caused by mere heat treatment. The experiment was designed into four groups: control (WT), control (*Nlrp3*^*-/-*^), no LPS/heat (WT), and no LPS/heat (*Nlrp3*^*-/-*^). The comparison was conducted between *Nlrp3*^*-/-*^ and wild-type mice. Hypothalamus IL-1β, IL-6, and TNF-α was measured by ELISA. **a** Survival curve was monitored. The statistical analysis was conducted by Log-rank test (*n* = 20). **b** Core temperature (T_C_) profiles of mice were monitored in every 15 min. The average time taken by core temperature to reach 42 °C was calculated by mean ± SEM. (*n* = 5 to 10). **c** Tissue homogenate level of IL-1β was measured. **d** Tissue homogenate level of IL-6 was measured. **e** Tissue homogenate level of TNF-α was measured. Each cytokine concentration were expressed as the mean ± SEM. (*n* = 5 to 10). **f** Immunohistochemical result of IL-1β between control (WT) vs no LPS/Heat (WT) group mice. Three representative pictures were selected. **g** Quantification and statistical analysis of the immunohistochemical microscopies. (*n* = 5–10) **p < 0.01. ^ns^*p >* 0.05 (ns: no significance)

In order to determine the role of NLRP3 inflammasome in heat tolerance, the weight-matched *Nlrp3* knockout mice were also subjected to heat challenge with and without the presence of LPS. The mean survival time and inflammatory cytokines in the CNS were collected. First, we assessed the protective effect of *Nlrp3* knockout on Heat treatment alone without LPS. Regrettably, although the mean survival time of *Nlrp3*^-/-^ mice increased compared with WT mice, the difference was not significant (175.10 ± 18.23 min versus 167.78 ± 2.50 min respectively, Fig. 4a, log-rank *p* = 0.13). In the comparison of the mean time duration taken for T_C_ to increase from resting to 42 °C, there was also no significant finding (112 ± 21.34 min versus 110 ± 5.88 min, *p* = 0.89, Fig. 4b).

Regarding the level of neuroinflammation, IL-1β, IL-6, and TNF-α were measured in homogenized mice diencephalon tissue (mainly the hypothalamus) by using ELISA. Assessment of IL-1β showed that there was certainly significant difference between *Nlrp3*^-/-^ and WT mice (10.84 ± 2.30 pg/ml vs 21.82 ± 3.15 pg/ml, *p* < 0.01, Fig. 4c). However, in the context of IL-6 and TNF-α, we detected no significant difference between *Nlrp3*^-/-^and WT mice (Fig. 4d and e).

To further explore the effect of heat alone on neuroinflammation, IL-1β level between control (WT) vs no LPS/heat (WT) mice was further detected by using immunohistochemical analysis, the result found no significant difference between control and no LPS/heat mice after immunohistochemical microscopy quantification (Fig. 4f and g).

### *Nlrp3* knockout prolonged mice survival time in LPS/heat treatment by inhibiting IL-1β induced neuroinflammation

To further address whether *Nlrp3*knockout can protect mice from heat stroke, and based on the result of NLRP3 inflammasome activation in Fig. 3, *Nlrp3*^-/-^ mice was subjected to LPS/heat treatment in comparison with WT mice.

**Fig. 5:**
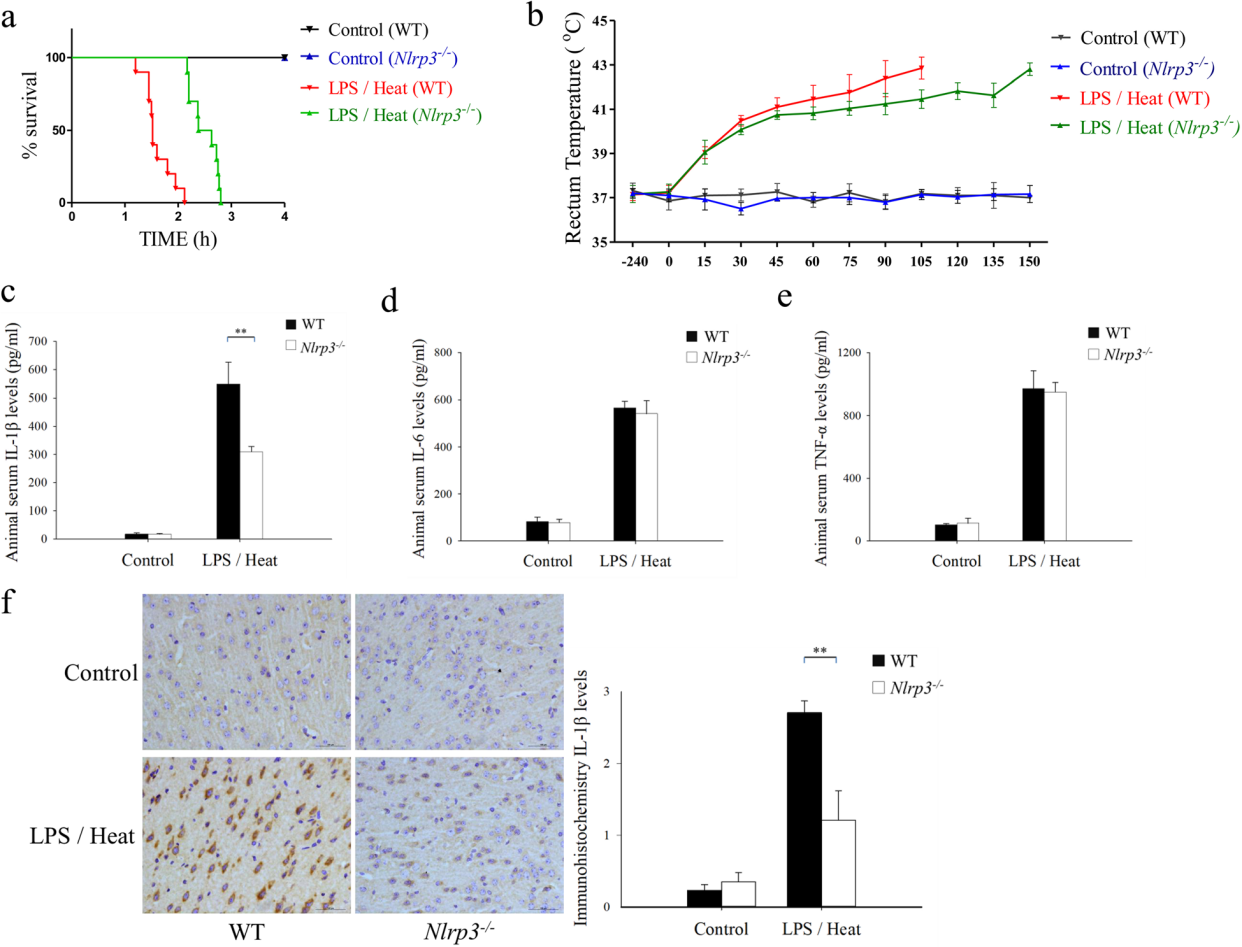
*Nlrp3* knockout protected mice from heat stroke-induced mice death by inhibiting IL-1β releasing. The experiment was designed into four groups: control (WT), control (*Nlrp3*^*-/-*^), LPS/heat (WT), and LPS/heat (*Nlrp3*^*-/-*^).LPS treatment condition was 1 mg/kg and whole-body heating (WBH) temperature was 41.2 °C. Systemic pro-inflammatory cytokines and hypothalamusIL-1β were measured by ELISA and immunohistochemistry, respectively. The comparison was conducted between *Nlrp3* knock out and wild-type mice. **a** Survival curve was monitored. The statistical analysis was conducted by Log-rank test (*n* = 20). **b** Core temperature (T_C_) profiles of mice were monitored in every 15 min. The average time taken by core temperature to reach 42 °C was calculated by mean ± SEM. (*n* = 5 to 10). **c** Serum level of pro-inflammatory cytokines IL-1β was measured by ELISA. **d** Serum level of pro-inflammatory cytokines IL-6 was measured by ELISA. **e** Serum level of pro-inflammatory cytokines TNF-α was measured by ELISA. **f** Hypothalamus IL-1β were measured immunohistochemistry, the H-score method was conducted for the staining quantitation. Each cytokine concentration and immunohistochemistry quantitation were expressed as the mean ± SEM. (*n* = 5 to 10). **p < 0.01, *p < 0.05

Without surprise, *Nlrp3* knockout was associated with significantly improved survival time under LPS/heat treatment compared with WT LPS/heat group (average survival time 96.66 ± 5.14 min versus 149.94 ± 4.94 min respectively, Fig. 5a, log-rank *p* < 0.01) in the survival study. The mean time duration taken for T_C_ to increase from resting to 42 °C was also significantly longer in *Nlrp3*^-/-^ mice (83.05 ± 6.15 min versus 134.34 ± 6.60 min, *p* < 0.01, Fig. 5b).

In order to further explore the underlying mechanism of prolonged survival time in *Nlrp3* knockout in LPS/heat mice, mice serum was collected and IL-1β, IL-6 and TNF-α were measured in Fig. 5. As expected, the result indicated significant reduced IL-1β in *Nlrp3*^-/-^ mice compared with WT mice under LPS/heat condition (549.05 ± 76.92 pg/ml vs 309.44 ± 19.14 pg/ml, *p* < 0.01, Fig. 5c). In contrast to IL-1β, there was no difference observed in IL-6 and TNF-α.

We then further explored the in situ IL-1β production in hypothalamus by using immunohistochemistry assay. The immunohistochemistry result was shown in Fig. 5f; after quantification of multiple histological sections, we found significantly decreased IL-1β production in *Nlrp3*^-/-^ mice hypothalamus.

### IL-1β neutralizing antibody inflated heat intolerance with the presence of prior infection

**Fig. 6:**
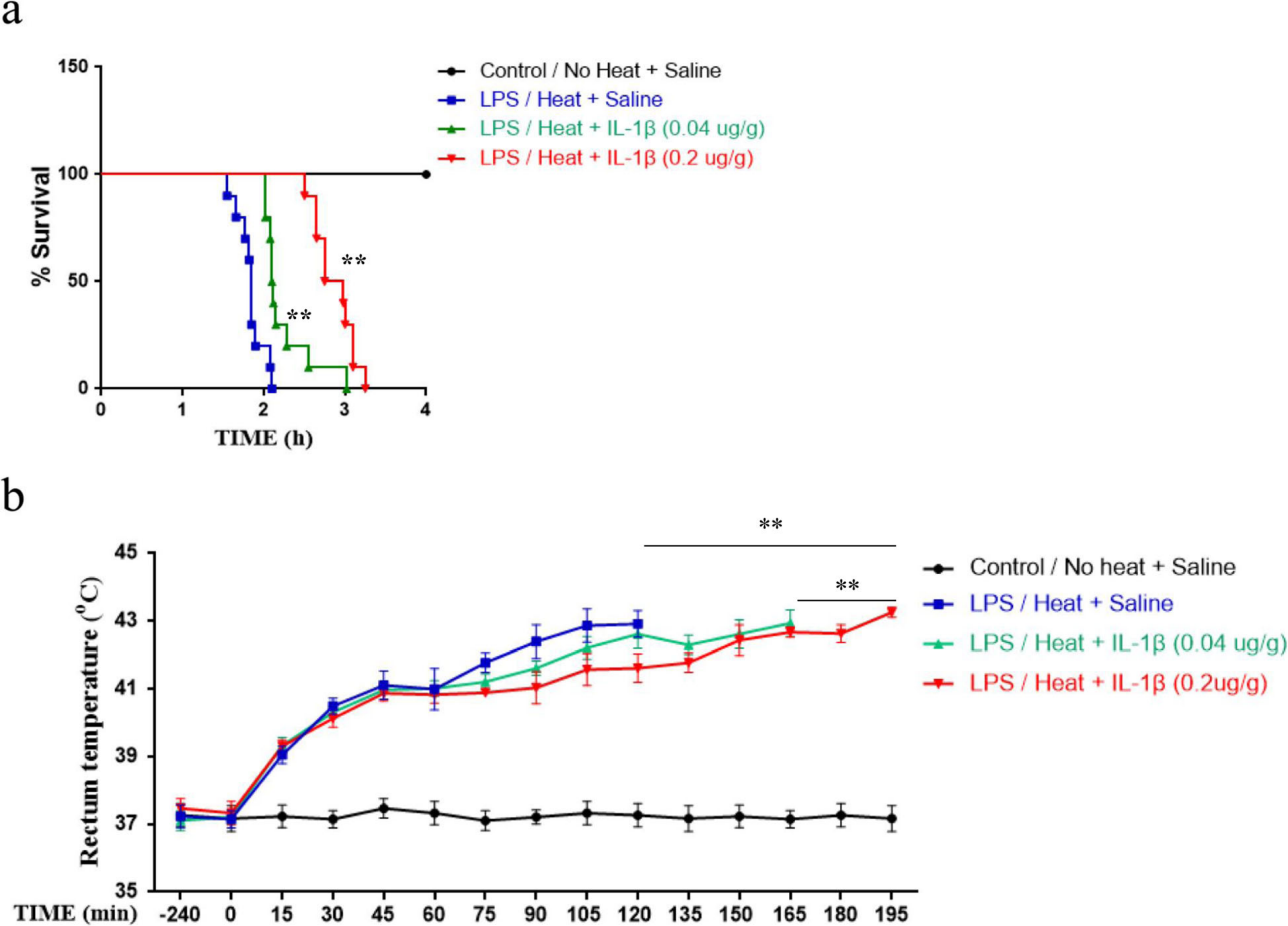
IL-1β neutralizing antibody enhanced heat intolerance induced by heat stroke. Two dosages of 0.04 μg/g and 0.2 μg/g IL-1β neutralizing antibody were injected intravenously 30 min before heat stroke. Saline was involved in the control. **a** Survival curve was monitored in comparison between saline and IL-1β neutralizing antibody groups. The statistical analysis was conducted by Log-rank test (*n* = 20), **p < 0.01, *p < 0.05. **b** Core temperature (T_C_) profiles of mice were monitored in every 15 min to compare saline and IL-1β neutralizing antibody groups. The average time taken by core temperature to reach 42 °C was calculated as the mean ± SEM. (*n* = 5 to 10). **p < 0.01

To further investigate the role of the IL-1β in LPS/heat induced damage, IL-1β neutralizing antibody was administrate just 30 min before heat. As shown, IL-1β neutralizing antibody was associated with significantly improved survival time after LPS/heat treatment with *p* < 0.01 in both 0.04 μg/g (134.64 ± 18.87 pg/ml vs 110.58 ± 10.02 pg/ml, *p* < 0.01, Fig. 6a) and 0.2 μg/g groups (172.38 ± 14.72 pg/ml vs 110.58 ± 10.02 pg/ml, *p* < 0.01, Fig. 6a) compared with saline. Furthermore, the mean duration taken for T_C_ to increase from resting to 42 °C was 105.67 ± 4.37 min vs 89.33 ± 27.86 min for 0.04 μg/g IL-1β neutralizing antibody group and 138.40 ± 17.98 min vs 89.33 ± 27.86 min for 0.2 μg/g IL-1β neutralizing antibody group (Fig. 6b). These heating durations were also significantly different when compared with saline group with *p* < 0.01 for each comparison.

**Fig. 7:**
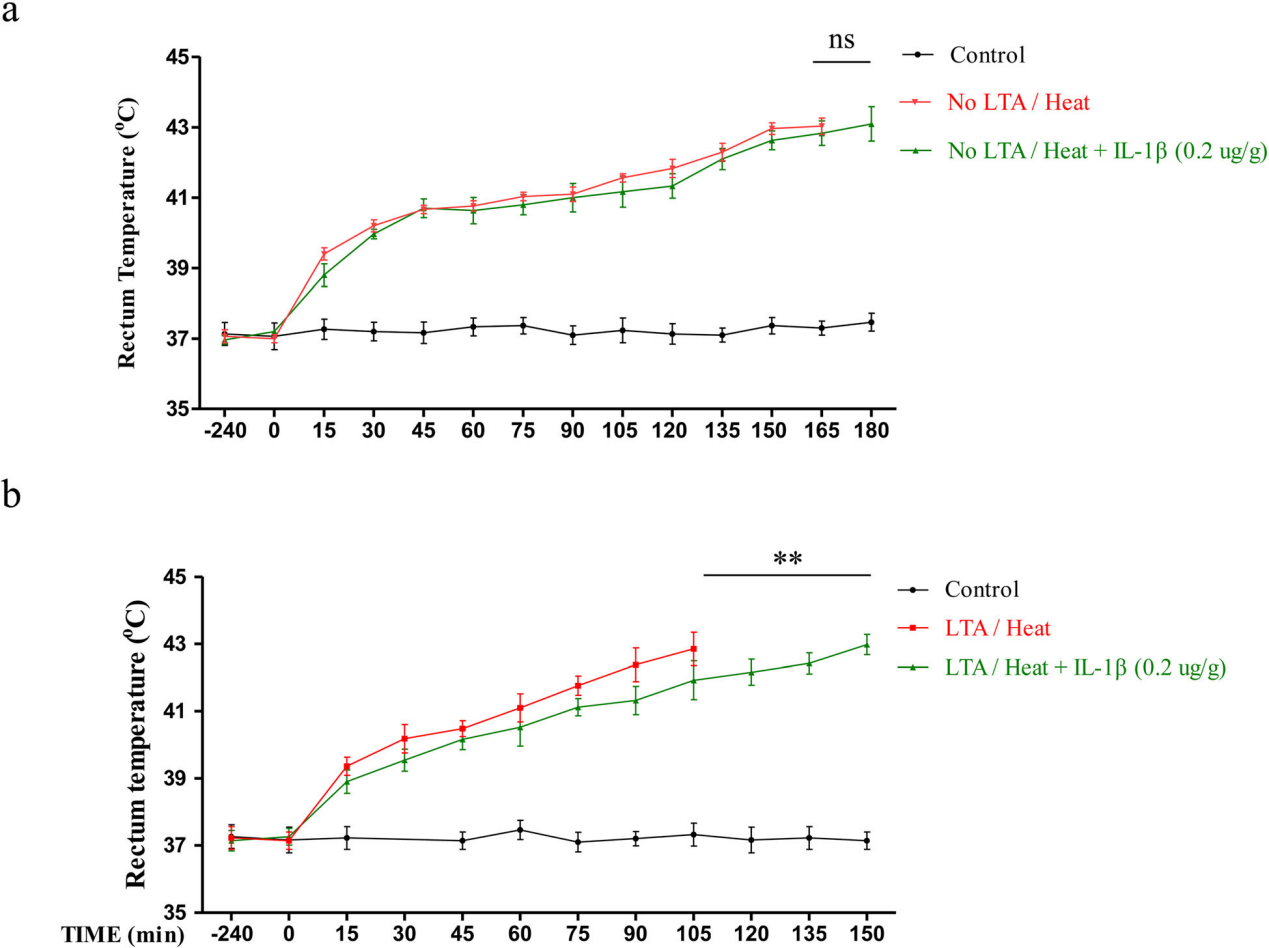
IL-1β neutralizing antibody failed to enhance heat intolerance under heat alone. Single dosages of 0.2 μg/g IL-1β neutralizing antibody were injected intravenously 30 min before heat stroke. Saline was involved in the control. **a** Core temperature (T_C_) profiles of mice were monitored in every 15 min to compare saline and IL-1β neutralizing antibody groups in the absence of prior infection. **b** Core temperature (T_C_) profiles of mice were monitored in every 15 min to compare saline and IL-1β neutralizing antibody groups with prior infection of LTA. The average time taken by core temperature to reach 42 °C was calculated as the mean ± SEM. (n = 5 to 10). **p < 0.01, ^ns^*p* > 0.05 (ns: no significance)

To further test the anti-IL-1β effect, we explored IL-1β response in the absence of prior infection. Our result indicated that although anti-IL-1β therapy (0.2μg/g) could extend the time it took for the animal’s body temperature to rise to 42 °C (132.04 ± 11.32 min vs 120.00 ± 15.03 min) without prior infection, the difference was not significant (*p* = 0.2, Fig. 7a). Furthermore, we explored the result of IL-1β antibody treatment in LTA/heat group. Similar to the result of LPS, the result found that anti-IL-1β therapy could significant slowdown the speed of animal body temperature rise (84.80 ± 6.14 min vs 109.60 ± 10.98 min, *p* < 0.01, Fig. 7b).

## Discussion

Heat stroke is a severe condition clinically diagnosed as a body temperature elevation with CNS disorder including delirium, seizures, and coma. Because of the further climate change, heat waves are expected to be longer and more intense in the future and heat stroke-related diseases have drawn increasing attention from public health policies [25]. The risk of heat stroke increases dramatically and even to be life-threatening in immune which compromises individuals with pre-existing illness, cardiovascular disease, drug use, and poor fitness level [26]. Besides from heat stroke itself, patients with latent inflammatory condition were more prone to heat stroke-induced injuries, and the injuries in these patients were often lethal [27].

During heat stroke, the blood is redistributed throughout the body during heat stress, leading to intestinal ischemic damage and increased permeability, which causes numerous intestinal G- bacilli to enter the blood and produce endotoxemia [28]. Heat stroke was commonly reported to compromise the epithelial junction integrity of intestinal mucosa and induce pro-inflammatory substances to leak into the circulating blood stream, which made heat stroke resembled sepsis in many aspects [29]. In addition to this, G+ bacteria caused infection, such as *Streptococcus pneumoniae* and *Staphylococcus aureus*, which represent a serious burden for infectious diseases worldwide, which are also commonly associated with heat stroke patients [30]. In summary, latent infection was a common accompanying factor during heat stroke.

Lipopolysaccharide (LPS) or lipoteichoic acid (LTA) treatment was commonly used to mimic infection and preexist inflammatory condition in heat stroke [13]. These facts triggered our interest to explore the underlining mechanism of heat stroke in susceptible individuals. In this study, we established a heat stroke animal model with prior infection, which we believe is a better simulation of clinical scenario in susceptible population.

In light of this, animals were divided into four groups, which were control/no heat, control/heat, LPS/no heat, and LPS/heat groups (control/no heat, control/heat, LTA/no heat, and LTA/heat groups) in our experiment. In LPS/LTA treatment, we deliberately chose a low dose (1 mg/kg) to mimic non-symptomatic prior infection compared with previous publication [13]. The results confirmed that low dose of LPS/LTA used here (1 mg/kg) itself made no difference in the survival analysis. Moreover, such a low dosage even had no impact on animals’ T_C_ change (Fig. 1b). However, LPS treatment could cause an increase in IL-1β, IL-6, and TNF-α in peripheral circulation and LTA alone failed to induce animal serum IL-1β increase (Fig. 2). By contrast, heat treatment alone generated lethal effect on animals. But the lethal effect was independent on inflammatory level in either serum level or CNS level (Fig. 2, Fig. 3c, Fig. 7a). Our study indicated that the prior infection could largely amplify the lethal effect of heat stroke as shown in decreased survival time (Fig. 1a) and increased inflammatory cytokines (Fig. 2, Fig. 7b). These results together suggested a synergistic effect between prior infection and heat which were consistent with what was observed in clinical practice.

Infection and heat induce similar inflammatory responses when experienced in succession, and they have a synergistic effect on shared mechanisms leading to organ injury [31]. In our results, TNF-α and IL-6 were rapidly increased upon infection and heat stroke which was also previously reported [32]. As contrast, IL-1β was usually detected subsequently as it was not constitutively expressed in cell cytoplasm. When infection and heat stroke occurred simultaneously or in short succession, IL-1β was extensively produced. Similar synergistic effect was also explored as for the mechanism of increasing risk of heat stroke which related pathology following infection. Publication demonstrated coagulopathy might be a potential candidate for the mechanism since both heat and infection produced a coagulopathy dysfunction, which together create a positive feedback resulting in the collapse of the coagulation system [33, 34].

In this study, we first reported that NLRP3 inflammasome activation mediated neuroinflammation as a driving mechanistic force in heat stroke pathology, especially when it was exacerbated by prior infection.

The NLRP3 inflammasome is one of the most important components of the innate immunity of the body. NLRP3 can bind ASC and caspase-1 to form a complex that promotes the maturation of inflammatory factors such as IL-1β and IL-18 [35]. IL-1β is a single-chain polypeptide glycoprotein that is mainly secreted by macrophages or monocytes. Studies have shown that the mortality of heat stroke was closely related to the level of IL-1β [15]. The injection of IL-1β in rats causes symptoms such as low blood pressure, visceral vasoconstriction, and decreased cardiac output, which are similar to the vital signs in rats with heatstroke [36]. In this study, we found that short-term heat stoke alone failed to activate the NLRP3 inflammasome, whereas heat stroke in combination with even minor LPS pretreatment greatly activated NLRP3 inflammasome in mice hypothalamus which was further confirmed by immunofluorescence staining assay (Fig. 3g).

So as to comprehensively understanding the role of NLRP3 inflammasome in heat stroke, *Nlrp3* knockout mice were used. We first examined its role in heat treatment alone. To our surprise, though *Nlrp3*^-/-^ mice demonstrated significantly reduced IL-1β level in the hypothalamus tissue. The *Nlrp3*^-/-^ mice failed to demonstrate any advantage in mean survival time or time duration taken to 42 °C (Fig. 4a and b). Evidence supported for the null effect might be heat alone was incapable to induce NLRP3 activation as shown in Fig. 3. This result further demonstrated heat stroke to induce organ and tissue damage in a NLRP3-independent mechanism. Conversely, *Nlrp3* knockout enhanced mice heat tolerance and extended survival time in LPS/heat group by decreasing both systemic and hypothalamus IL-1β production, whereas the IL-6 and TNF-α levels in these mice were not different from those of the wild-type mice (Fig. 5).

Furthermore, we found that the administration of IL-1β receptor neutralizing antibodies significantly slowed down T_C_ elevation and prolonged mice survival time under heatstroke with prior infection (Figs. 6 and 7b). In contrast to this result, in the absence of prior infection, anti-IL-1β failed to demonstrate an exact therapeutic effect (Fig. 7a). These results, together with other study reporting the participation of IL-1β in heat stroke, provided strong evidence of the role of NLRP3/IL-1β-mediated neuroinflammation as a main driving force in heat stroke pathology under prior infection and indicated alternative mechanism for pure heat stress. Regardless of the difference, such result proved that relevant IL-1β inhibition may be a therapy choice, which has been proven effective in a broad spectrum of diseases [37].

NLRP3 inflammasome upregulation and activation has been found to be associated in the development of many major diseases such as gout, type 2 diabetes, obesity-induced insulin resistance, and depression [38, 39]. In particular, the NLRP3 inflammasome is activated in response to cellular stresses through a two-component pathway, involving Toll-like receptor 4-ligand interaction (priming) and followed by a second signal, such as ATP-dependent P2X7 receptor activation [40]. The first signal leads to the production of IL-1β and IL-18 precursors and the second signal refers to inflammasome assembly and cleaves of pro-caspase-1 to its active form Caspase-1 p10, which further cleaves pro-IL-1β to IL-1β. Our team has been interested in the potential role of physical stimuli in NLRP3 inflammasome activation [19, 41]. Based on that, we then hypothesized that heat was likely served as the second signal for the activation and maturation of the inflammasome. Then, the hypothesis was confirmed by showing that either LPS or heat stroke alone failed to induce significant caspase-1 p10 or IL-1β maturation. Interestingly, when LPS administration preceded heat, NLRP3 inflammasome was strongly activated and IL-1β was secreted in the mice diencephalon (Fig. 3). This result provided evidence that heat stroke was capable to induce NLRP3 inflammasome assembly and was functionally similar with the second signal.

Taken together, our findings suggest when heat and infection experienced together; the two inflammation-promoting stimuli produced a synergistic effect, instigating an uncontrollable neuroinflammation to generate thermoregulation crash and incipient death.

## Conclusions

In summary, this study demonstrated the heat stroke pathophysiology in mice with prior infection. By using NLRP3 gene knockout mice, this study for the first time revealed the involvement of CNS NLRP3 inflammasome activation in above mechanism by alleviating heat stroke which induced hypothalamic neuronal inflammation. These findings not only increased the understanding of the mechanism in heat stroke injuries, but also provided a therapeutic target for the prevention and treatment of heatstroke in immune-compromised individuals. Moreover, this is also the first study reporting the capability of physical stimulation acting as the second signal in NLRP3 inflammasome activation which greatly deepened on our understanding of innate immune system.

## Data Availability

The data generated during our study cannot be publicly available due to the data safety concern. But the data are available from the corresponding author on reasonable request.

## Abbreviations

CNS: Central nervous system
T_C_: Core temperature
NLRP3: NLR family pyrin domain containing 3
LPS: Lipopolysaccharides
LTA: Lipoteichoic acid
IL-1β: Interleukin-1β
IL-6: Interleukin-6
TNF-α: Tumor necrosis factor-α
ELISA: Enzyme-linked immunosorbent assay
LDH: Lactate dehydrogenase
WT: Wild type
